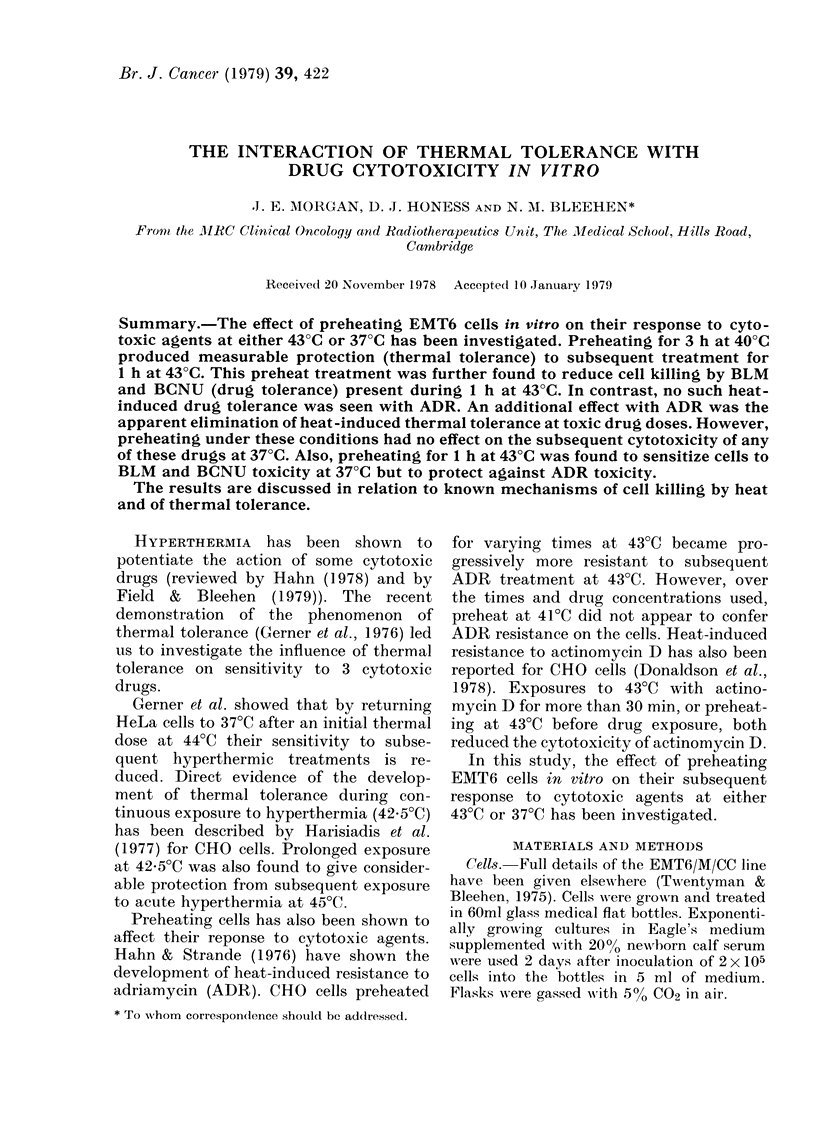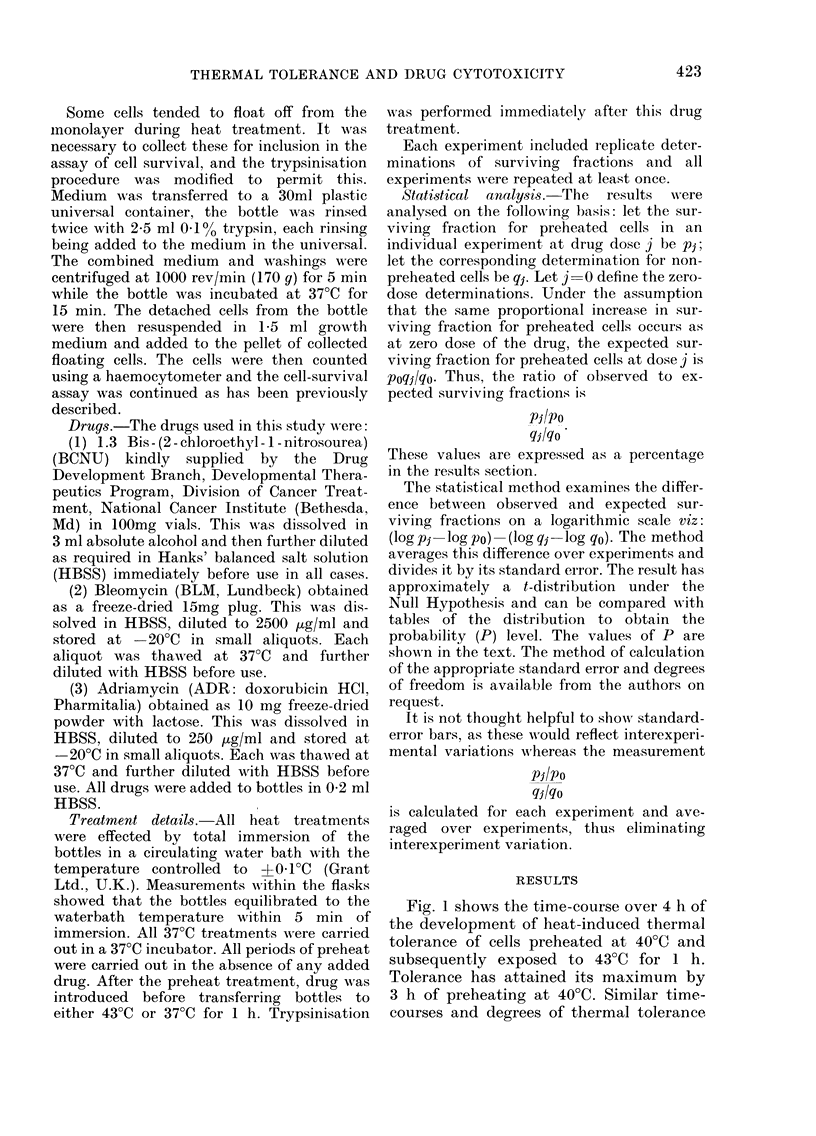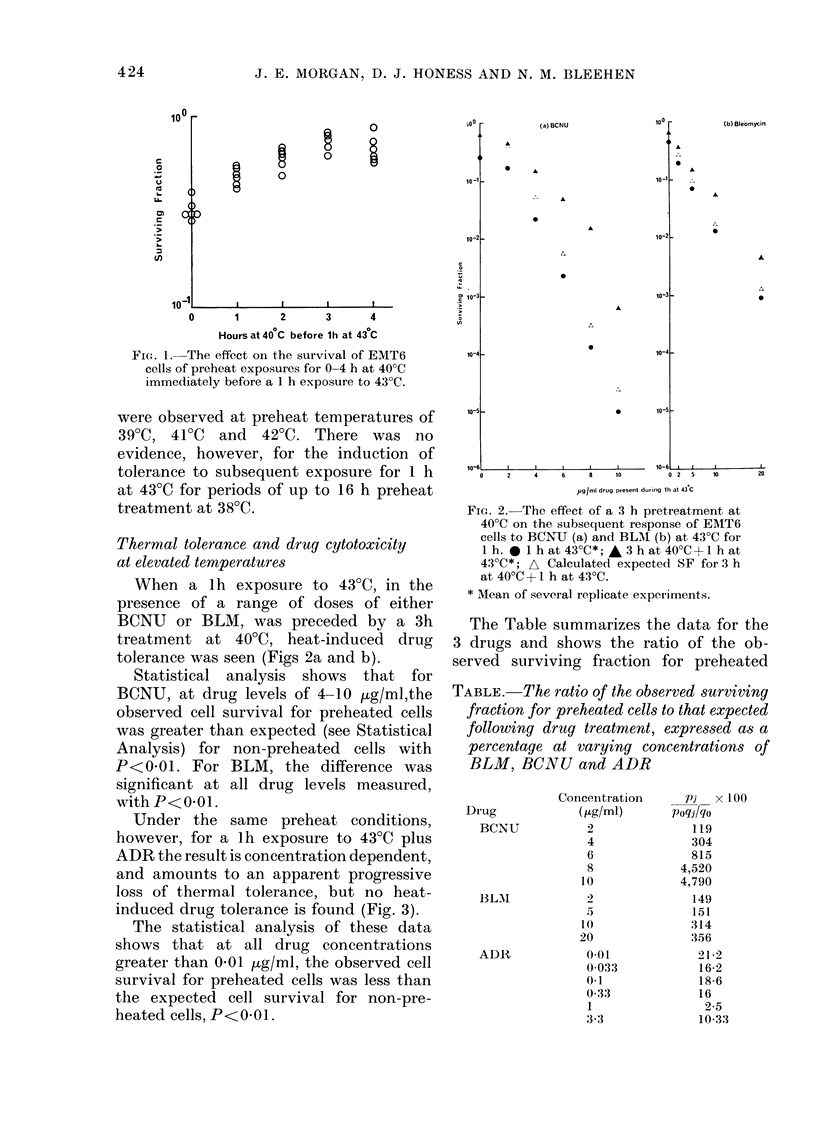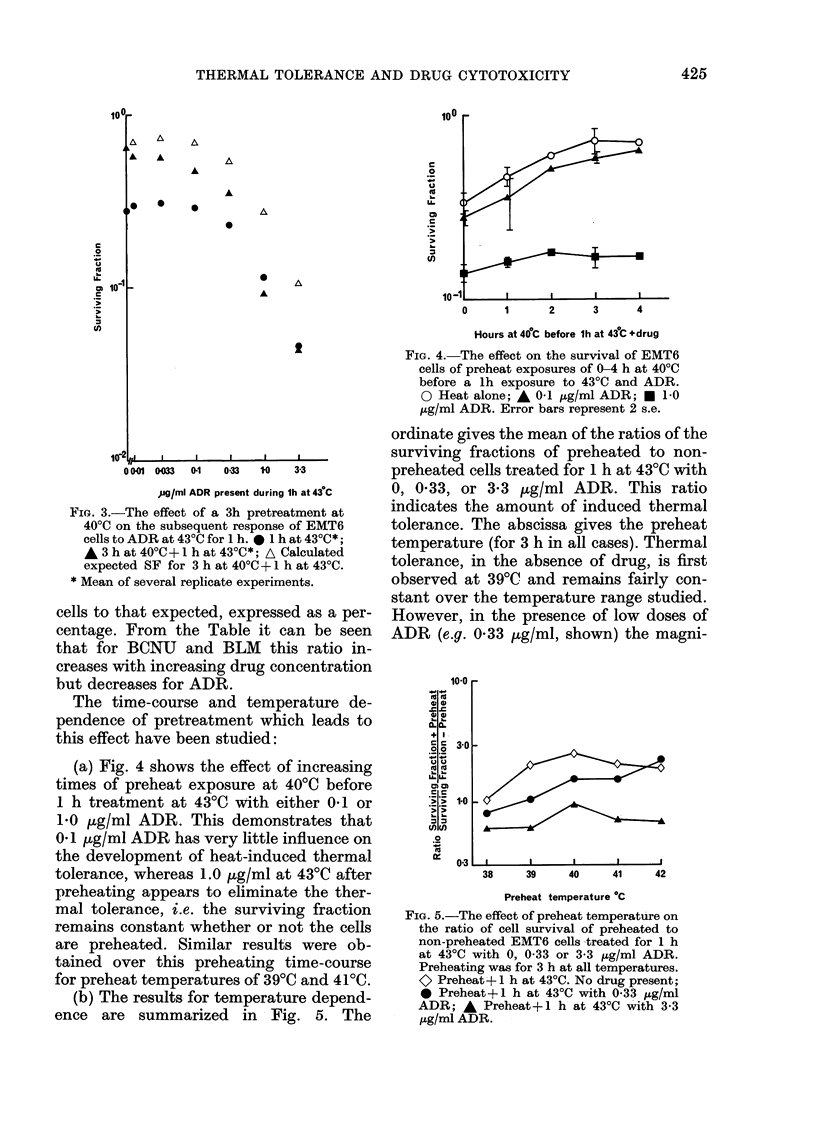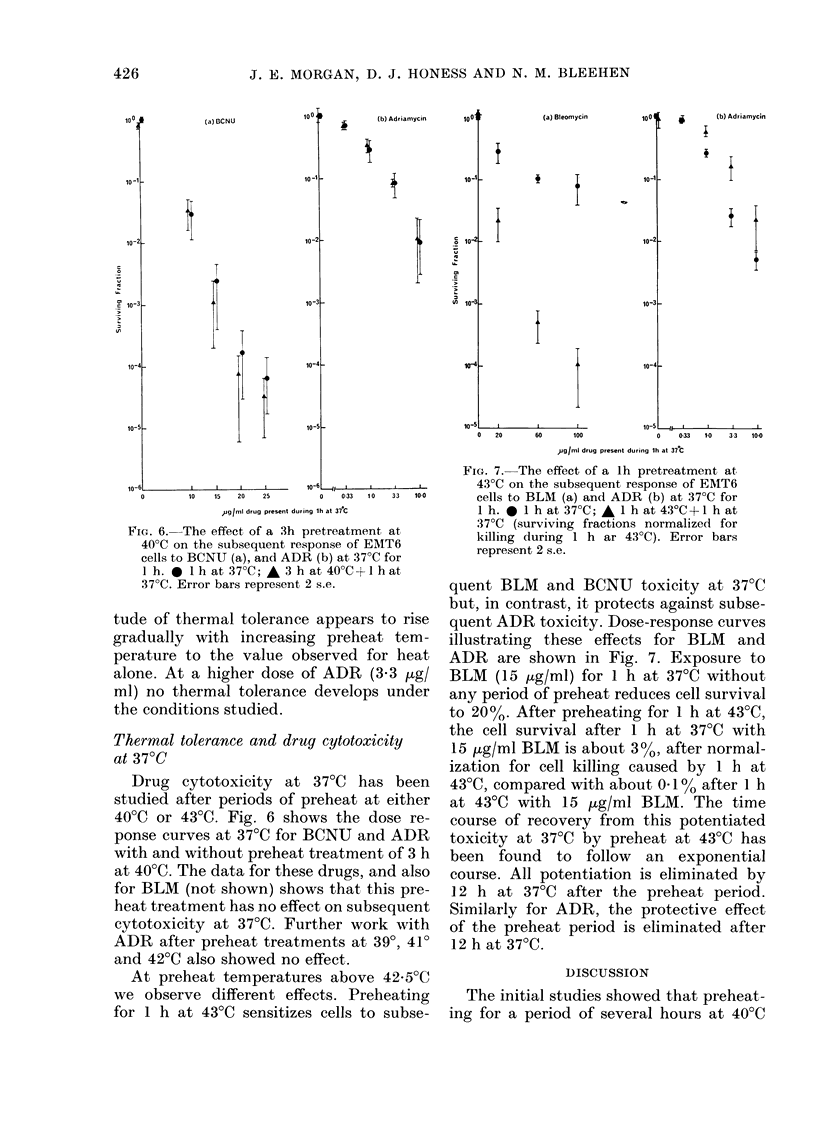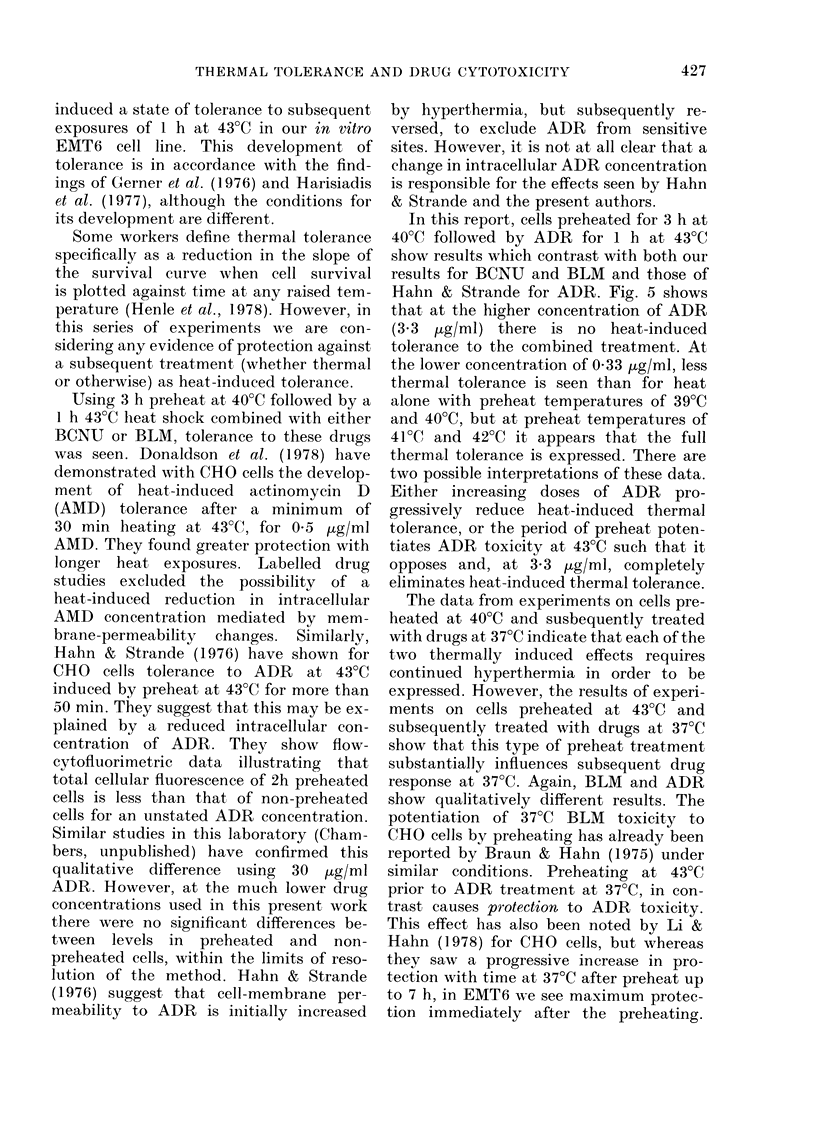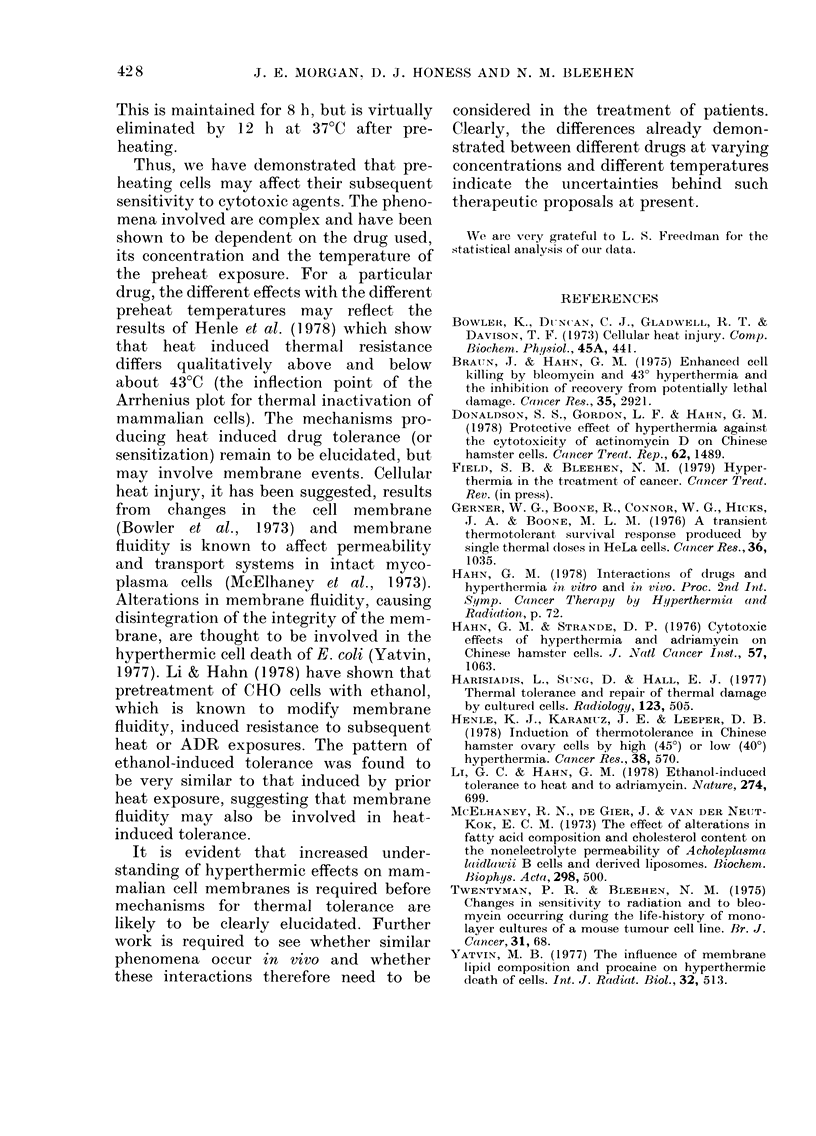# The interaction of thermal tolerance with drug cytotoxicity in vitro.

**DOI:** 10.1038/bjc.1979.75

**Published:** 1979-04

**Authors:** J. E. Morgan, D. J. Honess, N. M. Bleehen

## Abstract

The effect of preheating EMT6 cells in vitro on their response to cytotoxic agents of either 43 degrees C or 37 degrees C has been investigated. Preheating for 3 h at 40 degrees C produced measurable protection (thermal tolerance) to subsequent treatment for 1 h at 43 degrees C. This preheat treatment was further found to reduce cell killing by BLM and BCNU (drug tolerance) present during 1 h at 43 degrees C. In contrast, no such heat-induced drug tolerance was seen with ADR. An additional effect with ADR was the apparent elimination of heat-induced thermal tolerance at toxic drug doses. However, preheating under these conditions had no effect on the subsequent cytotoxicity of any of these drugs at 37 degrees C. Also, preheating for 1 h at 43 degrees C was found to sensitize cells to BLM and BCNU toxicity at 37 degrees C but to protect against ADR toxicity. The results are discussed in relation to known mechanisms of cell killing by heat and of thermal tolerance.


*Br. J. Cancer* (1979) **39**, 422

### THE INTERACTION OF THERMAL TOLERANCE WITH DRUG CYTOTOXICITY *IN VITRO*

J. E. MORGAN, D. J. HONESS AND N. M. BLEEHEN*

*From the MRC Clinical Oncology and Radiotherapeutics Unit, The Medical School, Hills Road, Cambridge*

Received 20 November 1978 Accepted 10 January 1979

**Summary.—The effect of preheating EMT6 cells *in vitro* on their response to cytotoxic agents at either 43°C or 37°C has been investigated. Preheating for 3 h at 40°C produced measurable protection (thermal tolerance) to subsequent treatment for 1 h at 43°C. This preheat treatment was further found to reduce cell killing by BLM and BCNU (drug tolerance) present during 1 h at 43°C. In contrast, no such heat-induced drug tolerance was seen with ADR. An additional effect with ADR was the apparent elimination of heat-induced thermal tolerance at toxic drug doses. However, preheating under these conditions had no effect on the subsequent cytotoxicity of any of these drugs at 37°C. Also, preheating for 1 h at 43°C was found to sensitize cells to BLM and BCNU toxicity at 37°C but to protect against ADR toxicity.**

**The results are discussed in relation to known mechanisms of cell killing by heat and of thermal tolerance.**

HYPERTHERMIA has been shown to potentiate the action of some cytotoxic drugs (reviewed by Hahn (1978) and by Field & Bleehen (1979)). The recent demonstration of the phenomenon of thermal tolerance (Gerner *et al.*, 1976) led us to investigate the influence of thermal tolerance on sensitivity to 3 cytotoxic drugs.

Gerner *et al.* showed that by returning HeLa cells to 37°C after an initial thermal dose at 44°C their sensitivity to subsequent hyperthermic treatments is reduced. Direct evidence of the development of thermal tolerance during continuous exposure to hyperthermia (42·5°C) has been described by Harisiadis *et al.* (1977) for CHO cells. Prolonged exposure at 42·5°C was also found to give considerable protection from subsequent exposure to acute hyperthermia at 45°C.

Preheating cells has also been shown to affect their reponse to cytotoxic agents. Hahn & Strande (1976) have shown the development of heat-induced resistance to adriamycin (ADR). CHO cells preheated for varying times at 43°C became progressively more resistant to subsequent ADR treatment at 43°C. However, over the times and drug concentrations used, preheat at 41°C did not appear to confer ADR resistance on the cells. Heat-induced resistance to actinomycin D has also been reported for CHO cells (Donaldson *et al.*, 1978). Exposures to 43°C with actinomycin D for more than 30 min, or preheating at 43°C before drug exposure, both reduced the cytotoxicity of actinomycin D.

In this study, the effect of preheating EMT6 cells *in vitro* on their subsequent response to cytotoxic agents at either 43°C or 37°C has been investigated.

### MATERIALS AND METHODS

*Cells.*—Full details of the EMT6/M/CC line have been given elsewhere (Twentyman & Bleehen, 1975). Cells were grown and treated in 60ml glass medical flat bottles. Exponentially growing cultures in Eagle's medium supplemented with 20% newborn calf serum were used 2 days after inoculation of $2 \times 10^5$ cells into the bottles in 5 ml of medium. Flasks were gassed with 5% $CO_2$ in air.

* To whom correspondence should be addressed.

Some cells tended to float off from the monolayer during heat treatment. It was necessary to collect these for inclusion in the assay of cell survival, and the trypsinisation procedure was modified to permit this. Medium was transferred to a 30ml plastic universal container, the bottle was rinsed twice with 2·5 ml 0·1% trypsin, each rinsing being added to the medium in the universal. The combined medium and washings were centrifuged at 1000 rev/min (170 *g*) for 5 min while the bottle was incubated at 37°C for 15 min. The detached cells from the bottle were then resuspended in 1·5 ml growth medium and added to the pellet of collected floating cells. The cells were then counted using a haemocytometer and the cell-survival assay was continued as has been previously described.

*Drugs*.—The drugs used in this study were:

(1) 1.3 Bis-(2-chloroethyl-1-nitrosourea) (BCNU) kindly supplied by the Drug Development Branch, Developmental Therapeutics Program, Division of Cancer Treatment, National Cancer Institute (Bethesda, Md) in 100mg vials. This was dissolved in 3 ml absolute alcohol and then further diluted as required in Hanks' balanced salt solution (HBSS) immediately before use in all cases.

(2) Bleomycin (BLM, Lundbeck) obtained as a freeze-dried 15mg plug. This was dissolved in HBSS, diluted to 2500 μg/ml and stored at −20°C in small aliquots. Each aliquot was thawed at 37°C and further diluted with HBSS before use.

(3) Adriamycin (ADR: doxorubicin HCl, Pharmitalia) obtained as 10 mg freeze-dried powder with lactose. This was dissolved in HBSS, diluted to 250 μg/ml and stored at −20°C in small aliquots. Each was thawed at 37°C and further diluted with HBSS before use. All drugs were added to bottles in 0·2 ml HBSS.

*Treatment details*.—All heat treatments were effected by total immersion of the bottles in a circulating water bath with the temperature controlled to ±0·1°C (Grant Ltd., U.K.). Measurements within the flasks showed that the bottles equilibrated to the waterbath temperature within 5 min of immersion. All 37°C treatments were carried out in a 37°C incubator. All periods of preheat were carried out in the absence of any added drug. After the preheat treatment, drug was introduced before transferring bottles to either 43°C or 37°C for 1 h. Trypsinisation was performed immediately after this drug treatment.

Each experiment included replicate determinations of surviving fractions and all experiments were repeated at least once.

*Statistical analysis*.—The results were analysed on the following basis: let the surviving fraction for preheated cells in an individual experiment at drug dose $j$ be $p_j$; let the corresponding determination for non-preheated cells be $q_j$. Let $j=0$ define the zero-dose determinations. Under the assumption that the same proportional increase in surviving fraction for preheated cells occurs as at zero dose of the drug, the expected surviving fraction for preheated cells at dose $j$ is $p_0 q_j / q_0$. Thus, the ratio of observed to expected surviving fractions is

$$\frac{p_j/p_0}{q_j/q_0}.$$

These values are expressed as a percentage in the results section.

The statistical method examines the difference between observed and expected surviving fractions on a logarithmic scale *viz*: $(\log p_j - \log p_0) - (\log q_j - \log q_0)$. The method averages this difference over experiments and divides it by its standard error. The result has approximately a $t$-distribution under the Null Hypothesis and can be compared with tables of the distribution to obtain the probability ($P$) level. The values of $P$ are shown in the text. The method of calculation of the appropriate standard error and degrees of freedom is available from the authors on request.

It is not thought helpful to show standard-error bars, as these would reflect interexperimental variations whereas the measurement

$$\frac{p_j/p_0}{q_j/q_0}$$

is calculated for each experiment and averaged over experiments, thus eliminating interexperiment variation.

### RESULTS

Fig. 1 shows the time-course over 4 h of the development of heat-induced thermal tolerance of cells preheated at 40°C and subsequently exposed to 43°C for 1 h. Tolerance has attained its maximum by 3 h of preheating at 40°C. Similar time-courses and degrees of thermal tolerance

FIG. 1.—The effect on the survival of EMT6 cells of preheat exposures for 0–4 h at 40°C immediately before a 1 h exposure to 43°C.

FIG. 2.—The effect of a 3 h pretreatment at 40°C on the subsequent response of EMT6 cells to BCNU (a) and BLM (b) at 43°C for 1 h. ● 1 h at 43°C*; ▲ 3 h at 40°C + 1 h at 43°C*; △ Calculated expected SF for 3 h at 40°C + 1 h at 43°C.

* Mean of several replicate experiments.

were observed at preheat temperatures of 39°C, 41°C and 42°C. There was no evidence, however, for the induction of tolerance to subsequent exposure for 1 h at 43°C for periods of up to 16 h preheat treatment at 38°C.

*Thermal tolerance and drug cytotoxicity at elevated temperatures*

When a 1h exposure to 43°C, in the presence of a range of doses of either BCNU or BLM, was preceded by a 3h treatment at 40°C, heat-induced drug tolerance was seen (Figs 2a and b).

Statistical analysis shows that for BCNU, at drug levels of 4–10 μg/ml,the observed cell survival for preheated cells was greater than expected (see Statistical Analysis) for non-preheated cells with $P<0{\cdot}01$. For BLM, the difference was significant at all drug levels measured, with $P<0{\cdot}01$.

Under the same preheat conditions, however, for a 1h exposure to 43°C plus ADR the result is concentration dependent, and amounts to an apparent progressive loss of thermal tolerance, but no heat-induced drug tolerance is found (Fig. 3).

The statistical analysis of these data shows that at all drug concentrations greater than 0·01 μg/ml, the observed cell survival for preheated cells was less than the expected cell survival for non-preheated cells, $P<0{\cdot}01$.

The Table summarizes the data for the 3 drugs and shows the ratio of the observed surviving fraction for preheated

TABLE.—*The ratio of the observed surviving fraction for preheated cells to that expected following drug treatment, expressed as a percentage at varying concentrations of BLM, BCNU and ADR*

| Drug | Concentration (μg/ml) | $\frac{p_j}{p_0 q_j / q_0} \times 100$ |
|---|---|---|
| BCNU | 2 | 119 |
| | 4 | 304 |
| | 6 | 815 |
| | 8 | 4,520 |
| | 10 | 4,790 |
| BLM | 2 | 149 |
| | 5 | 151 |
| | 10 | 314 |
| | 20 | 356 |
| ADR | 0·01 | 21·2 |
| | 0·033 | 16·2 |
| | 0·1 | 18·6 |
| | 0·33 | 16 |
| | 1 | 2·5 |
| | 3·3 | 10·33 |

FIG. 3.—The effect of a 3h pretreatment at 40°C on the subsequent response of EMT6 cells to ADR at 43°C for 1 h. ● 1 h at 43°C*; ▲ 3 h at 40°C+1 h at 43°C*; △ Calculated expected SF for 3 h at 40°C+1 h at 43°C.

* Mean of several replicate experiments.

FIG. 4.—The effect on the survival of EMT6 cells of preheat exposures of 0–4 h at 40°C before a 1h exposure to 43°C and ADR. ○ Heat alone; ▲ 0·1 μg/ml ADR; ■ 1·0 μg/ml ADR. Error bars represent 2 s.e.

cells to that expected, expressed as a percentage. From the Table it can be seen that for BCNU and BLM this ratio increases with increasing drug concentration but decreases for ADR.

The time-course and temperature dependence of pretreatment which leads to this effect have been studied:

(a) Fig. 4 shows the effect of increasing times of preheat exposure at 40°C before 1 h treatment at 43°C with either 0·1 or 1·0 μg/ml ADR. This demonstrates that 0·1 μg/ml ADR has very little influence on the development of heat-induced thermal tolerance, whereas 1.0 μg/ml at 43°C after preheating appears to eliminate the thermal tolerance, *i.e.* the surviving fraction remains constant whether or not the cells are preheated. Similar results were obtained over this preheating time-course for preheat temperatures of 39°C and 41°C.

(b) The results for temperature dependence are summarized in Fig. 5. The ordinate gives the mean of the ratios of the surviving fractions of preheated to non-preheated cells treated for 1 h at 43°C with 0, 0·33, or 3·3 μg/ml ADR. This ratio indicates the amount of induced thermal tolerance. The abscissa gives the preheat temperature (for 3 h in all cases). Thermal tolerance, in the absence of drug, is first observed at 39°C and remains fairly constant over the temperature range studied. However, in the presence of low doses of ADR (*e.g.* 0·33 μg/ml, shown) the magni-

FIG. 5.—The effect of preheat temperature on the ratio of cell survival of preheated to non-preheated EMT6 cells treated for 1 h at 43°C with 0, 0·33 or 3·3 μg/ml ADR. Preheating was for 3 h at all temperatures. ◇ Preheat+1 h at 43°C. No drug present; ● Preheat+1 h at 43°C with 0·33 μg/ml ADR; ▲ Preheat+1 h at 43°C with 3·3 μg/ml ADR.

FIG. 6.—The effect of a 3h pretreatment at 40°C on the subsequent response of EMT6 cells to BCNU (a), and ADR (b) at 37°C for 1 h. ● 1 h at 37°C; ▲ 3 h at 40°C+1 h at 37°C. Error bars represent 2 s.e.

FIG. 7.—The effect of a 1h pretreatment at 43°C on the subsequent response of EMT6 cells to BLM (a) and ADR (b) at 37°C for 1 h. ● 1 h at 37°C; ▲ 1 h at 43°C+1 h at 37°C (surviving fractions normalized for killing during 1 h ar 43°C). Error bars represent 2 s.e.

tude of thermal tolerance appears to rise gradually with increasing preheat temperature to the value observed for heat alone. At a higher dose of ADR (3·3 μg/ml) no thermal tolerance develops under the conditions studied.

*Thermal tolerance and drug cytotoxicity at 37°C*

Drug cytotoxicity at 37°C has been studied after periods of preheat at either 40°C or 43°C. Fig. 6 shows the dose reponse curves at 37°C for BCNU and ADR with and without preheat treatment of 3 h at 40°C. The data for these drugs, and also for BLM (not shown) shows that this preheat treatment has no effect on subsequent cytotoxicity at 37°C. Further work with ADR after preheat treatments at 39°, 41° and 42°C also showed no effect.

At preheat temperatures above 42·5°C we observe different effects. Preheating for 1 h at 43°C sensitizes cells to subsequent BLM and BCNU toxicity at 37°C but, in contrast, it protects against subsequent ADR toxicity. Dose-response curves illustrating these effects for BLM and ADR are shown in Fig. 7. Exposure to BLM (15 μg/ml) for 1 h at 37°C without any period of preheat reduces cell survival to 20%. After preheating for 1 h at 43°C, the cell survival after 1 h at 37°C with 15 μg/ml BLM is about 3%, after normalization for cell killing caused by 1 h at 43°C, compared with about 0·1% after 1 h at 43°C with 15 μg/ml BLM. The time course of recovery from this potentiated toxicity at 37°C by preheat at 43°C has been found to follow an exponential course. All potentiation is eliminated by 12 h at 37°C after the preheat period. Similarly for ADR, the protective effect of the preheat period is eliminated after 12 h at 37°C.

### DISCUSSION

The initial studies showed that preheating for a period of several hours at 40°C

induced a state of tolerance to subsequent exposures of 1 h at 43°C in our *in vitro* EMT6 cell line. This development of tolerance is in accordance with the findings of Gerner *et al.* (1976) and Harisiadis *et al.* (1977), although the conditions for its development are different.

Some workers define thermal tolerance specifically as a reduction in the slope of the survival curve when cell survival is plotted against time at any raised temperature (Henle *et al.*, 1978). However, in this series of experiments we are considering any evidence of protection against a subsequent treatment (whether thermal or otherwise) as heat-induced tolerance.

Using 3 h preheat at 40°C followed by a 1 h 43°C heat shock combined with either BCNU or BLM, tolerance to these drugs was seen. Donaldson *et al.* (1978) have demonstrated with CHO cells the development of heat-induced actinomycin D (AMD) tolerance after a minimum of 30 min heating at 43°C, for 0·5 $\mu$g/ml AMD. They found greater protection with longer heat exposures. Labelled drug studies excluded the possibility of a heat-induced reduction in intracellular AMD concentration mediated by membrane-permeability changes. Similarly, Hahn & Strande (1976) have shown for CHO cells tolerance to ADR at 43°C induced by preheat at 43°C for more than 50 min. They suggest that this may be explained by a reduced intracellular concentration of ADR. They show flow-cytofluorimetric data illustrating that total cellular fluorescence of 2h preheated cells is less than that of non-preheated cells for an unstated ADR concentration. Similar studies in this laboratory (Chambers, unpublished) have confirmed this qualitative difference using 30 $\mu$g/ml ADR. However, at the much lower drug concentrations used in this present work there were no significant differences between levels in preheated and non-preheated cells, within the limits of resolution of the method. Hahn & Strande (1976) suggest that cell-membrane permeability to ADR is initially increased by hyperthermia, but subsequently reversed, to exclude ADR from sensitive sites. However, it is not at all clear that a change in intracellular ADR concentration is responsible for the effects seen by Hahn & Strande and the present authors.

In this report, cells preheated for 3 h at 40°C followed by ADR for 1 h at 43°C show results which contrast with both our results for BCNU and BLM and those of Hahn & Strande for ADR. Fig. 5 shows that at the higher concentration of ADR (3·3 $\mu$g/ml) there is no heat-induced tolerance to the combined treatment. At the lower concentration of 0·33 $\mu$g/ml, less thermal tolerance is seen than for heat alone with preheat temperatures of 39°C and 40°C, but at preheat temperatures of 41°C and 42°C it appears that the full thermal tolerance is expressed. There are two possible interpretations of these data. Either increasing doses of ADR progressively reduce heat-induced thermal tolerance, or the period of preheat potentiates ADR toxicity at 43°C such that it opposes and, at 3·3 $\mu$g/ml, completely eliminates heat-induced thermal tolerance.

The data from experiments on cells preheated at 40°C and susbequently treated with drugs at 37°C indicate that each of the two thermally induced effects requires continued hyperthermia in order to be expressed. However, the results of experiments on cells preheated at 43°C and subsequently treated with drugs at 37°C show that this type of preheat treatment substantially influences subsequent drug response at 37°C. Again, BLM and ADR show qualitatively different results. The potentiation of 37°C BLM toxicity to CHO cells by preheating has already been reported by Braun & Hahn (1975) under similar conditions. Preheating at 43°C prior to ADR treatment at 37°C, in contrast causes *protection* to ADR toxicity. This effect has also been noted by Li & Hahn (1978) for CHO cells, but whereas they saw a progressive increase in protection with time at 37°C after preheat up to 7 h, in EMT6 we see maximum protection immediately after the preheating.

This is maintained for 8 h, but is virtually eliminated by 12 h at 37°C after preheating.

Thus, we have demonstrated that preheating cells may affect their subsequent sensitivity to cytotoxic agents. The phenomena involved are complex and have been shown to be dependent on the drug used, its concentration and the temperature of the preheat exposure. For a particular drug, the different effects with the different preheat temperatures may reflect the results of Henle *et al.* (1978) which show that heat induced thermal resistance differs qualitatively above and below about 43°C (the inflection point of the Arrhenius plot for thermal inactivation of mammalian cells). The mechanisms producing heat induced drug tolerance (or sensitization) remain to be elucidated, but may involve membrane events. Cellular heat injury, it has been suggested, results from changes in the cell membrane (Bowler *et al.*, 1973) and membrane fluidity is known to affect permeability and transport systems in intact mycoplasma cells (McElhaney *et al.*, 1973). Alterations in membrane fluidity, causing disintegration of the integrity of the membrane, are thought to be involved in the hyperthermic cell death of *E. coli* (Yatvin, 1977). Li & Hahn (1978) have shown that pretreatment of CHO cells with ethanol, which is known to modify membrane fluidity, induced resistance to subsequent heat or ADR exposures. The pattern of ethanol-induced tolerance was found to be very similar to that induced by prior heat exposure, suggesting that membrane fluidity may also be involved in heat-induced tolerance.

It is evident that increased understanding of hyperthermic effects on mammalian cell membranes is required before mechanisms for thermal tolerance are likely to be clearly elucidated. Further work is required to see whether similar phenomena occur *in vivo* and whether these interactions therefore need to be considered in the treatment of patients. Clearly, the differences already demonstrated between different drugs at varying concentrations and different temperatures indicate the uncertainties behind such therapeutic proposals at present.

We are very grateful to L. S. Freedman for the statistical analysis of our data.

### REFERENCES

BOWLER, K., DUNCAN, C. J., GLADWELL, R. T. & DAVISON, T. F. (1973) Cellular heat injury. *Comp. Biochem. Physiol.*, **45A**, 441.

BRAUN, J. & HAHN, G. M. (1975) Enhanced cell killing by bleomycin and 43° hyperthermia and the inhibition of recovery from potentially lethal damage. *Cancer Res.*, **35**, 2921.

DONALDSON, S. S., GORDON, L. F. & HAHN, G. M. (1978) Protective effect of hyperthermia against the cytotoxicity of actinomycin D on Chinese hamster cells. *Cancer Treat. Rep.*, **62**, 1489.

FIELD, S. B. & BLEEHEN, N. M. (1979) Hyperthermia in the treatment of cancer. *Cancer Treat. Rev.* (in press).

GERNER, W. G., BOONE, R., CONNOR, W. G., HICKS, J. A. & BOONE, M. L. M. (1976) A transient thermotolerant survival response produced by single thermal doses in HeLa cells. *Cancer Res.*, **36**, 1035.

HAHN, G. M. (1978) Interactions of drugs and hyperthermia *in vitro* and *in vivo*. *Proc. 2nd Int. Symp. Cancer Therapy by Hyperthermia and Radiation*, p. 72.

HAHN, G. M. & STRANDE, D. P. (1976) Cytotoxic effects of hyperthermia and adriamycin on Chinese hamster cells. *J. Natl Cancer Inst.*, **57**, 1063.

HARISIADIS, L., SUNG, D. & HALL, E. J. (1977) Thermal tolerance and repair of thermal damage by cultured cells. *Radiology*, **123**, 505.

HENLE, K. J., KARAMUZ, J. E. & LEEPER, D. B. (1978) Induction of thermotolerance in Chinese hamster ovary cells by high (45°) or low (40°) hyperthermia. *Cancer Res.*, **38**, 570.

LI, G. C. & HAHN, G. M. (1978) Ethanol-induced tolerance to heat and to adriamycin. *Nature*, **274**, 699.

MCELHANEY, R. N., DE GIER, J. & VAN DER NEUT-KOK, E. C. M. (1973) The effect of alterations in fatty acid composition and cholesterol content on the nonelectrolyte permeability of *Acholeplasma laidlawii* B cells and derived liposomes. *Biochem. Biophys. Acta*, **298**, 500.

TWENTYMAN, P. R. & BLEEHEN, N. M. (1975) Changes in sensitivity to radiation and to bleomycin occurring during the life-history of monolayer cultures of a mouse tumour cell line. *Br. J. Cancer*, **31**, 68.

YATVIN, M. B. (1977) The influence of membrane lipid composition and procaine on hyperthermic death of cells. *Int. J. Radiat. Biol.*, **32**, 513.